# Bcl-2 Enhances Chimeric Antigen Receptor T Cell Persistence by Reducing Activation-Induced Apoptosis

**DOI:** 10.3390/cancers13020197

**Published:** 2021-01-08

**Authors:** Haiyong Wang, Ping Han, Xinyue Qi, Fanlin Li, Min Li, Lilv Fan, Huihui Zhang, Xiaoqing Zhang, Xuanming Yang

**Affiliations:** 1 Sheng Yushou Center of Cell Biology and Immunology, School of Life Sciences and Biotechnology, Shanghai Jiao Tong University, Shanghai 200240, China; picturechina@sjtu.edu.cn (H.W.); esterhanping@sjtu.edu.cn (P.H.); xinyueqi@sjtu.edu.cn (X.Q.); lifanlin@sjtu.edu.cn (F.L.); limin_1216@sjtu.edu.cn (M.L.); fanlilv@sjtu.edu.cn (L.F.); ninxia0@sjtu.edu.cn (H.Z.); zxq0318@sjtu.edu.cn (X.Z.); 2 Joint International Research Laboratory of Metabolic & Developmental Sciences, Shanghai Jiao Tong University, Shanghai 200240, China; 3 Key Laboratory of Systems Biomedicine (Ministry of Education), Shanghai Center for Systems Biomedicine, Shanghai Jiao Tong University, Shanghai 200240, China

**Keywords:** Bcl-2, CAR-T, lymphoma, CD20, immunotherapy

## Abstract

**Simple Summary:**

Chimeric antigen receptor-modified T cells (CAR-T) have shown great success in the treatment of B-cell leukemia. However, their efficacy is compromised in B-cell-derived lymphoma and solid tumors. Optimization of CAR design to improve in vivo persistence is a focus of current CAR-T cell research. The aim of our study is to access the potential added value of integration of anti-apoptotic molecules for enhancing anti-tumor activity of CAR-T cells. We confirmed that integrating B cell lymphoma-2 (Bcl-2) into CAR-T cells improved the proliferation ability of CAR-T cells in vitro and in vivo, which led to enhanced anti-tumor activity and prolonged survival in a mouse xenograft lymphoma model. This work provides proof of concept evidence for a new strategy to optimize the function of CAR-T cells against lymphoma.

**Abstract:**

Purpose: To evaluate the potential added value of integrating anti-apoptotic molecules for improving the anti-tumor activity of CAR-T cells. Methods: Four small molecules inhibiting apoptosis were tested for their ability to prevent activated induced CAR-T cell death. Five CD20-targeting, CD137 (4-1BB) and CD3ζ integrated CAR-T cells (20BBZ) with constitutively expressed anti-apoptotic genes were established, and we screened out the strongest proliferation enhancer: Bcl-2. The memory subtype and the exhaustion markers of CAR-T cells were analyzed. The anti-tumor activities of Bcl-2 integrating CAR-T cells (20BBZ-Bcl-2) were evaluated in vitro and in a mouse xenograft lymphoma model. Conclusion: The 20BBZ-Bcl-2 CAR-T cells showed improved proliferation ability compared to 20BBZ CAR-T cells in vitro. In addition, activation-induced apoptosis was reduced in the 20BBZ-Bcl-2 CAR-T cells. Consistent with the enhanced proliferation in vitro, 20BBZ-Bcl-2 CAR-T cells exhibited improved anti-tumor activity in a mouse xenograft lymphoma model.

## 1. Introduction

Chimeric antigen receptor (CAR) T cell therapy combines the specific reorganization of antibodies with the powerful intrinsic cytotoxic ability of T cells, which has been a revolutionary breakthrough for cancer immunotherapy [1]. CAR-T cell therapy has achieved a complete remission rate of ~80% in treating CD19^+^ relapsed/refractory aggressive B-cell leukemia and complete remission rates of ~30–60% for relapsed/refractor B-cell lymphoma [2,3]. However, its efficacy for solid tumors is greatly compromised. There are multiple challenges in treating solid tumors with CAR-T cell therapy, such as insufficient infiltration of CAR-T cells to tumor sites, poor T cell persistence, development of T cell exhaustion, and reduced effector function in immune suppressive tumor microenvironments. Novel approaches overcoming these limitations are urgently needed for CAR-T therapy against solid tumors [4,5,6].

Improving the persistence of CAR-T cells is critical for its in vivo efficacy, which is co-determined by the proliferation ability and the cell death of the CAR-T cells. Strategies have been developed to promote T cell proliferation, such as integration of Janus kinase-signal transducer and activator of transcription(JAK-STAT) signal into the CAR design [7], addition of a third cytokine signal [8], and providing additional co-stimulation signals [9]. Activation-induced cell death (AICD) mediates the contraction of antigen-activated T cells during T cell activation, which plays important roles in maintaining homeostasis of T cells, avoiding auto immune responses, and controlling the magnitude of T cell responses [10]. Repetitive engagement of the T cell receptor (TCR) in vitro stimulates T cells to upregulate Fas ligand (FasL) expression, which is followed by its binding to Fas in T cells to induce AICD [11]. AICD can be effectively blocked by inhibiting the CD95/CD95L pathway [10]. Whether additional survival-promoting molecules would be able to reduce AICD and enhance CAR-T cell persistence has not been investigated.

In the current study, we added the anti-apoptotic molecule Bcl-2 to a 4-1BB and CD3ζ (BBZ)-based second-generation CAR structure (20BBZ CAR-T cells) targeting CD20 to establish a novel CAR structure (20BBZ-Bcl-2 CAR-T cells). Compared with 20BBZ CAR-T cells, 20BBZ-Bcl-2 CAR-T cells exhibited reduced apoptosis, enhanced proliferation, and increased tendency in central memory phenotype in vitro. Furthermore, 20BBZ-Bcl-2 CAR-T cells showed better in vivo tumor control in a lymphoma xenograft mouse model. This work provides evidence supporting a new strategy to optimize CAR-T cell persistence for treating lymphoma.

## 2. Results

### 2.1. Apoptosis Occurred during CAR-T Cell Activation

**Figure 1:**
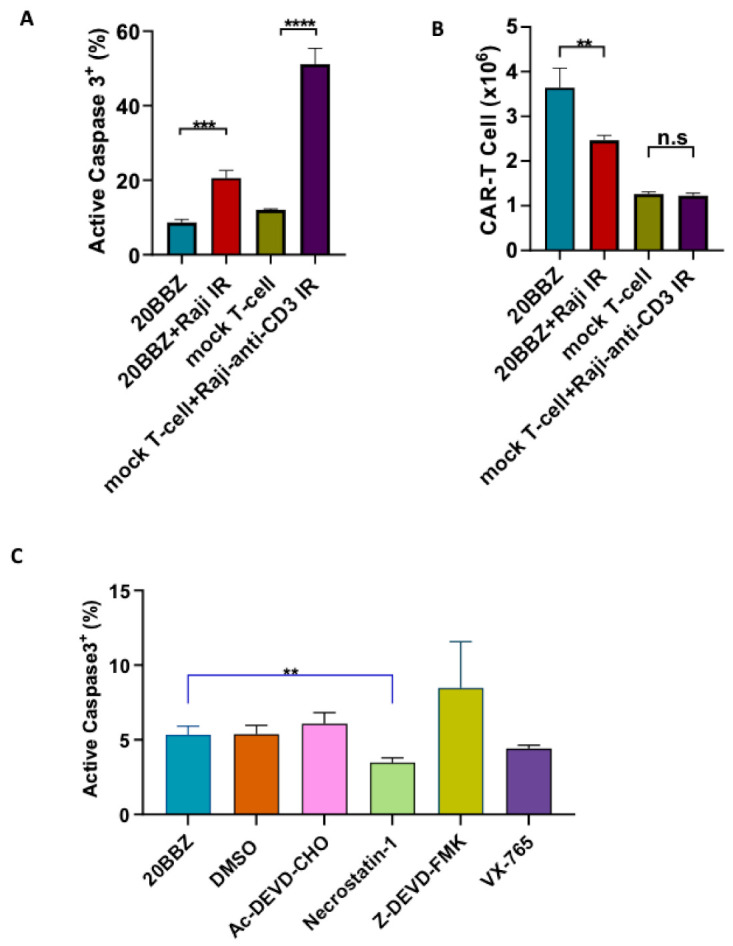
Inhibition of cell death enhanced chimeric antigen receptor-modified T cells (CAR-T) cell survival during activation. (**A**,**B**) CD20 targeting 20BBZ CAR-T cells or mock T cells were stimulated with irradiated CD20^+^ Raji cells, anti-CD3 expressing Raji cells, or were left unstimulated. Active caspase-3 was analyzed 24 h post stimulation by intracellular staining and flow cytometry (**A**). The numbers of live cells were counted and compared for each group (**B**). (**C**) 20BBZ CAR-T cells were stimulated with irradiated CD20^+^ Raji cell in the presence of the indicated cell death inhibitors. Active caspase-3 was analyzed 24 h later by intracellular staining and flow cytometry. Representative results of one from three replicate experiments are shown (**A**–**C**). Statistical significance was determined by unpaired t-test. Statistical significance was presented by ** *p* < 0.01, *** *p* < 0.001, **** *p* < 0.0001 and n.s ( not significant) (**A**–**C**).

Antigenic stimulation through the TCR promotes T cell proliferation but can also induce FasL-Fas interaction-mediated AICD. We wondered whether AICD occurred during CAR-T cell activation. To investigate this, we generated CD20 targeting CAR-T cells and stimulated them with CD20^+^ irradiated Raji cells. By staining for active caspase-3, we determined that antigenic stimulation of CAR-T cells significantly increased apoptosis (Figure 1A). Consistent with increased apoptosis, the number of live cells decreased significantly one day after the stimulation with irradiated Raji cells (Figure 1B). To test whether similar apoptosis occurred during TCR engagement, we stimulated mock T cells with irradiated anti-CD3 expressing Raji cells. After stimulation, mock T cells underwent apoptosis similar to CAR-T cells (Figure 1A), suggesting both CAR and TCR activation can induce T cell apoptosis. We then wondered whether the apoptosis could be reversed by small molecule inhibitors during CAR-T cell activation. Ac-DEVD-CHO and Z-DEVD-FMK are specific caspase-3 inhibitors [12,13]. Belnacasan (VX-765) is a selective caspase-1 inhibitor [14]. Necrostatin-1 can inhibit both Receptor-interacting Protein 1(RIP1) kinase and Indoleamine 2,3-Dioxygenase (IDO) [15]. We evaluated these cell death inhibitors targeting different pathways and found that nectrostatin-1 significantly reduced apoptosis during CAR-T activation, while the other inhibitors had little impact on apoptosis (Figure 1C). Collectively, these data suggested that targeting cell death can be a potential approach for improving CAR-T cell persistence.

### 2.2. Screening of Potential Survival Enhancing Molecules for CAR-T Cells

**Figure 2:**
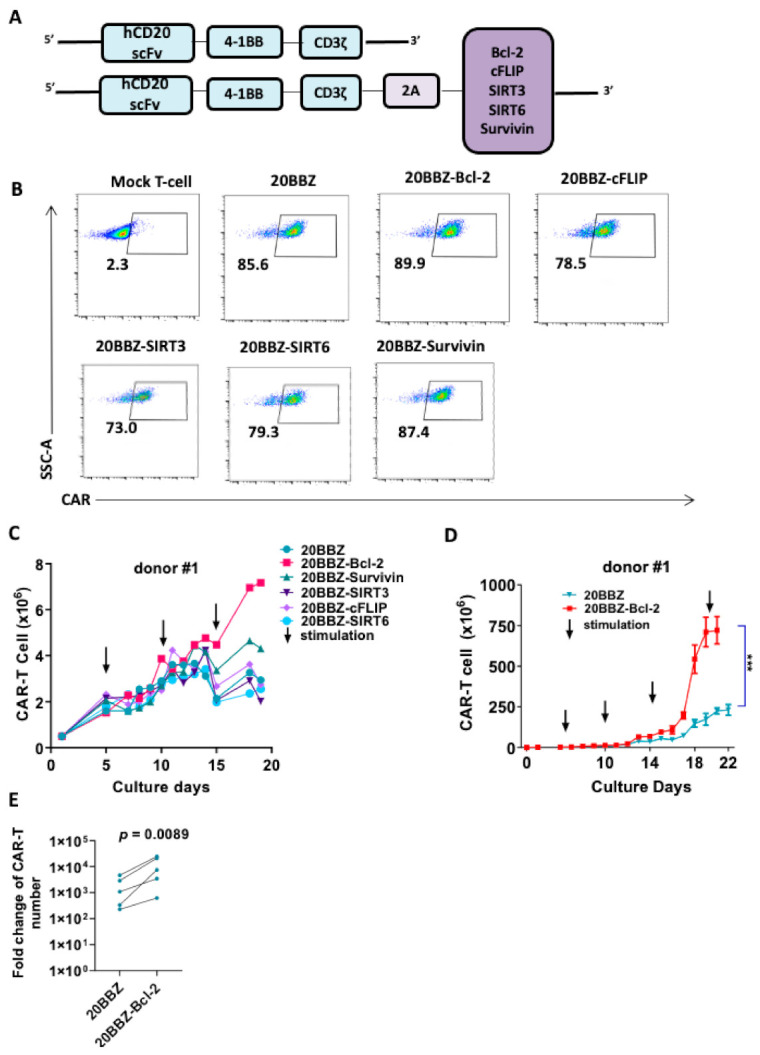
Bcl-2 as a potential enhancer of CAR-T cell survival. (**A**) Schematic diagram of the CD20- targeting CAR constructs used in the study. An anti-human CD20 single-chain variable fragment (scFv) was linked to 4-1BB and CD3ζ to generate the 20BBZ construct. Various survival-promoting molecules were linked to CD3ζ via a porcine teschovirus-1 2A (P2A) peptide. (**B**) Flow cytometry analysis of CAR expression on the indicated CAR-T cells. (**C**) Comparison of long-term proliferation of 20BBZ CAR-T cells and 20BBZ CAR-T cells with the indicated anti-apoptotic molecules. Arrows indicated the irradiated Raji stimulation (effector to target (E:T) = 3:1). (**D**) Overall expansion of CAR+ T cells in long-term cultures of CD20BBZ CAR-T cells and CD20BBZ-Bcl-2 CAR-T cells. Arrows indicated the irradiated Raji stimulation (E:T = 3:1). (**E**) 20BBZ and 20BBZ-Bcl-2 CAR-T cells proliferation on 22–28 days after long-term culture. Each dot presented one donor. Representative results from one of four replicate experiments are shown (**B**–**D**). Statistical significance was determined by unpaired t-test (**D**) or paired t-test (**E**). Statistical significance was presented by *** *p* < 0.001 or as indicated.

Although the cell death inhibitor necrostatin-1 reduced the apoptosis of CAR-T cell in vitro, this approach would not be suitable for in vivo cancer therapy. When used in vivo, these inhibitors could inhibit apoptosis in all cell types, including tumor cells. To overcome this limitation, we designed a series of CAR formats with integrated survival-promoting molecules (Figure 2A). Bcl-2 deficient mice demonstrate more lymphoid apoptosis [16], survivin is critical for OX40-meidated T cell clonal expansion [17], sirtuin 3 (SIRT3) activity promotes allogeneic donor T cell responses in allogeneic hematopoietic cell transplantation [18], cellular FADD-like interleukin-1β-converting enzyme (FLICE)-inhibitory protein (cFLIP) is required for T cell survival and cycling [19], and sirtuin 6 (SIRT6) protects against aging-associated pathologies by chromatin signaling and genome maintenance [20]. These molecules were linked to CAR using a cleavable 2A peptide. To evaluate whether the additional 2A-linked anti-apoptotic molecules affected the expression of CAR on the cell surface, we infected primary human T cells from three donors with the modified lentiviruses. The expression levels of the CARs were comparable, suggesting that the addition of the anti-apoptotic molecules did not affect CAR expression or cell-membrane localization (Figure 2B). Evaluation of these newly modified CAR-T cells for proliferation revealed that Bcl-2- and survivin-containing CAR-T cells exhibited enhanced proliferation after 13 days of culturing (Figure 2C and Figure S1). To determine the long-term survival-promoting ability of Bcl-2, we established a CAR-T cell culture protocol using weekly stimulation with irradiated Raji cells. Consistent with the short-term culture results, CAR-T cell proliferation and survival were greatly enhanced long-term when Bcl-2 was over expressed (Figure 2D,E and Figure S2). These data suggested that integrated anti-apoptotic molecules could provide long-lasting survival or proliferation benefits to CAR-T cells, which is an ideal option for in vivo cancer therapy. As Bcl-2 overexpressed CAR-T cells showed better proliferation capability than the other cells, we subsequently focused on these cells in our study.

### 2.3. Bcl-2 CAR-T Cells Showed Reduced Apoptosis after Activation

**Figure 3:**
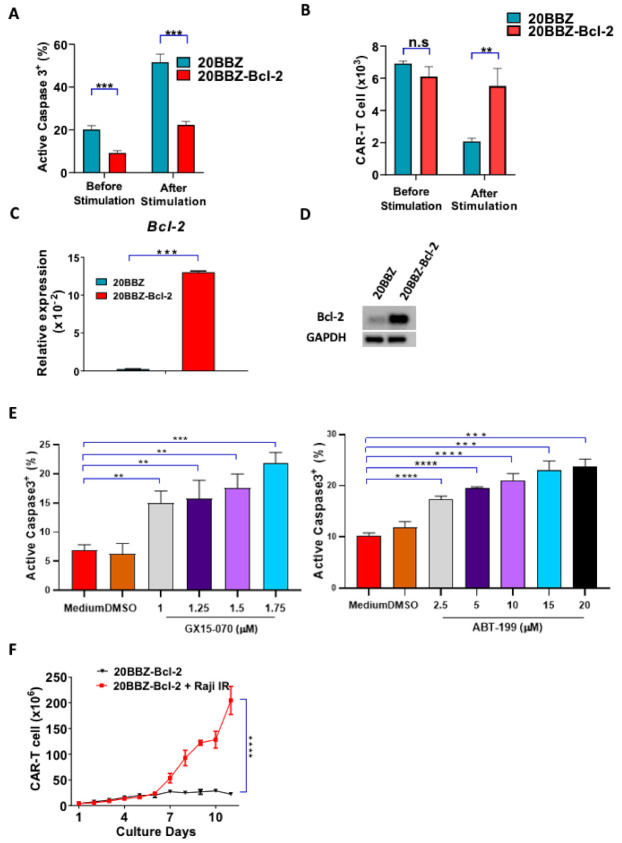
20BBZ-Bcl-2 CAR-T cells showed reduced apoptotic phenotype during long-term culturing. (**A**,**B**) 20BBZ CAR-T cells and 20BBZ-Bcl-2 CAR-T cells were stimulated with irradiated CD20^+^ Raji cells. Active caspase-3 was analyzed 24 h later by intracellular staining (**A**). The numbers of live cells were counted and compared for each group. (**B**) Representative results of one from three replicate experiments are shown. (**C**,**D**) The mRNA and the protein expression levels of Bcl-2 in 20BBZ and 20BBZ-Bcl-2 CAR-T cells were analyzed by real time (RT)-qPCR and western blot. Representative results of one from two replicate experiments are shown. (**E**) 20BBZ-Bcl-2 CAR-T cells were stimulated with irradiated CD20^+^ Raji cells in the presence of Bcl-2 inhibitors. Active caspase-3 was analyzed 24 h later by intracellular staining. Representative results of one from three replicate experiments are shown. (**F**) 20BBZ-Bcl-2 CAR-T cells were cultured with or without irradiated CD20^+^ Raji cells, and the number of live cells was counted at indicated time points. Representative results from one of three replicate experiments are shown. Statistical significance was determined by unpaired t-test. Statistical significance was presented by * *p* < 0.05, ** *p* < 0.01, *** *p* < 0.001, **** *p* < 0.0001 and n.s (not significant). (**A**–**C**,**E**,**F**).

Bcl-2 is an anti-apoptotic gene shown to have broad anti-apoptosis ability in various cell types [21]. We initially investigated whether Bcl-2 could affect AICD during CAR-T cell activation. We found that 20BBZ CAR-T cells showed a higher basal rate of apoptosis compared to that of the 20BBZ-Bcl-2 CAR-T cells. After antigen stimulation, more than 50% of the 20BBZ-CAR-T cells were apoptotic, while only 20% of the 20BBZ-Bcl-2 CAR-T cells showed an apoptotic phenotype (Figure 3A). Furthermore, inhibition of apoptosis enhanced 20BBZ-Bcl-2 CAR-T cell survival (Figure 3B). To determine whether this reduced apoptotic phenotype was directly mediated by Bcl-2, we detected mRNA expression levels of other important anti-apoptotic molecules, such as B-cell lymphoma-extralarge(Bcl-xl), Myeloid cell leukemia-1 (MCL-1), Bcl-2-like protein-2 (Bcl-w), and caspase-3, using quantitative reverse transcription polymerase chain reaction (RT-qPCR). Bcl-2 mRNA and protein were significantly enriched in the 20BBZ-Bcl-2 CAR-T cells (Figure 3C,D and Figure S3), suggesting Bcl-2 played a major role in reducing AICD. To confirm this, we treated 20BBZ-Bcl-2 CAR-T cells with two Bcl-2 inhibitors, GX15-070 and ABT-199. GX15-070 is a pan Bcl-2 family inhibitor, which inhibits specifically Bcl-xl, Bcl-2, MCL-1, Bcl-w, Bcl-2 related protein A1(Bfl-1) and BCL-2-like protein-10 (Bcl-B) [22]. ABT-199 is a more specific selective Bcl-2 inhibitor, which inhibits the growth of Bcl-2-dependent tumors [23]. When 20BBZ-Bcl-2 CAR-T cells were treated with the Bcl-2 inhibitors, apoptosis significantly increased (Figure 3D). Accordingly, we predicted that Bcl-2 could sustain long-term T cell persistence in the absence of antigen stimulation. To test this, we cultured 20BBZ-Bcl-2 CAR-T cells in the absence of antigen stimulation, which was designed to mimic the in vivo setting when the antigen was cleared by T cells. While our 20BBZ-Bcl-2 CAR-T cells did not expand, they persisted after 11 days of culturing without unrestricted growth (Figure 3E), suggesting a prolonged survival advantage. These data collectively indicated that Bcl-2 is a predominant anti-apoptotic regulatory protein during CAR-T cell activation, which provides a potential target for enhancing CAR-T cell survival.

### 2.4. Enhanced Anti-Apoptosis Capacity of Bcl-2 Did Not Affect CAR-T Cell Cytotoxicity Ability

**Figure 4:**
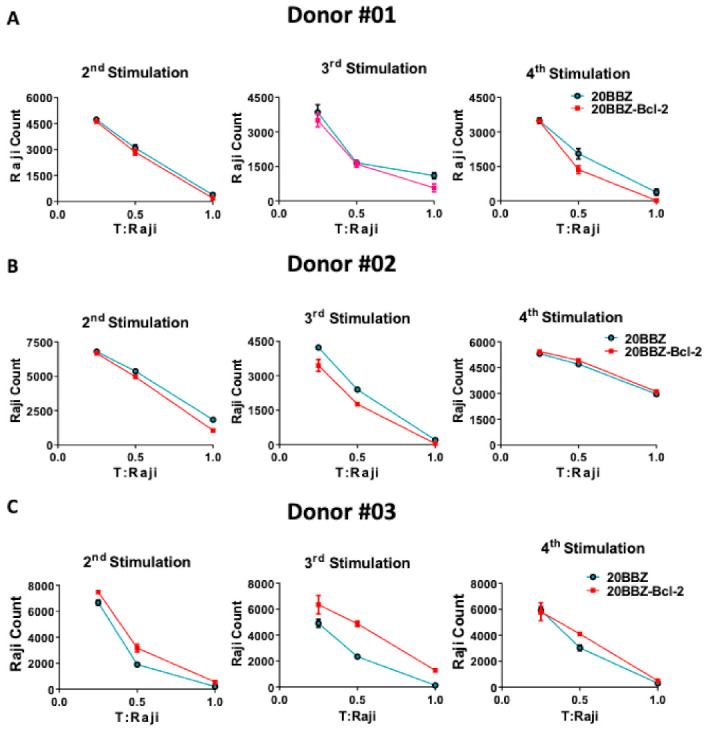
Cytotoxic ability profile of 20BBZ-Bcl-2 CAR-T cells in vitro. (**A**–**C**) Various donor-derived 20BBZ CAR-T cells and 20BBZ-Bcl-2 CAR-T cells at different culturing time points were co-cultured with Raji cells for 48 h (in triplicate) at an effector to target (E:T) ratios of 1:1, 1:2, and 1:4. Relative cytotoxicity was calculated by analyzing the remaining tumor cells (CD3^−^CD19^+^) using flow cytometry. Representative results from one of three replicate experiments are shown (**A**–**C**).

Cytotoxicity ability is the most important feature of CAR-T cells and directly determines their anti-tumor activity. To determine whether Bcl-2 signaling could enhance the cytotoxicity ability of CAR-T cells at different activation stages, we used repetitive antigen stimulation to mimic in vivo long-lasting chronic tumor burden. In this assay, T cells were re-stimulated every 6 days with irradiated CD20-positive Raji cells for a total of four times. Cells were harvested 4 days after each stimulation and incubated with live Raji cells at various T cell/tumor ratios (Figure 4A–C). To avoid the influence of percentage differences of CAR+ cells on cytotoxicity effect, we performed the tumor killing assay in vitro after the second round of stimulation with the irradiated Raji cells. After the second, the third, and the fourth stimulation with antigen, 20BBZ-Bcl-2 CAR-T cells exhibited similar cytotoxicity capabilities for various donors-derived CAR-T cells. These results suggested that, even though Bcl-2 significantly improved CAR-T cell survival, it had little impact on CAR-T cell cytotoxicity.

### 2.5. Bcl-2 Overexpression Altered 20BBZ CAR-T Cell Differentiation

**Figure 5:**
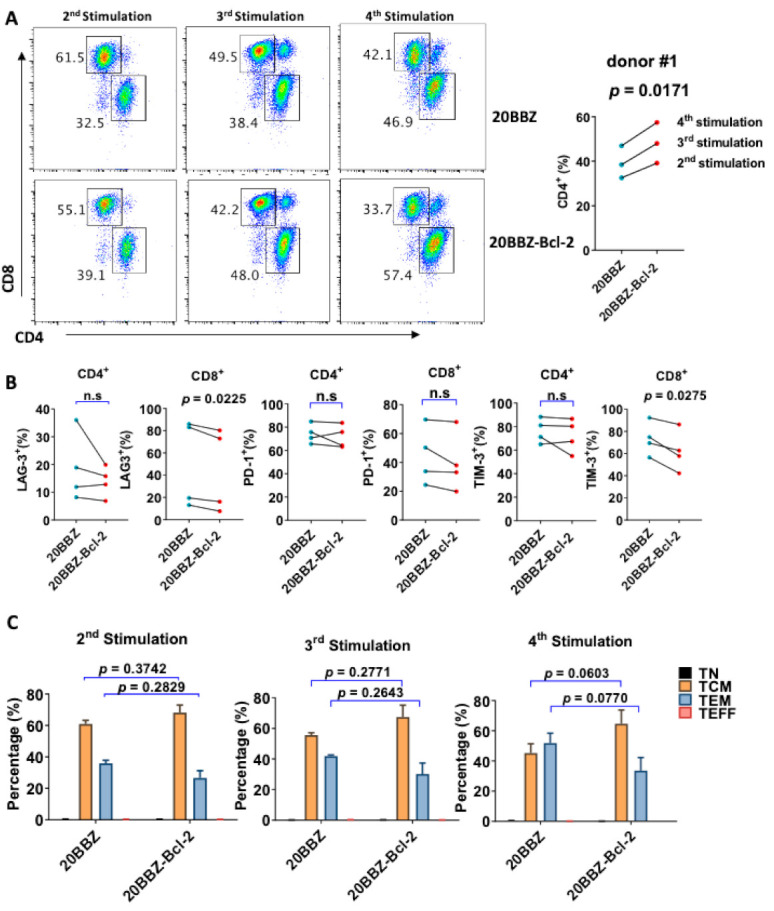
Bcl-2 signaling alters 20BBZ-Bcl-2 CAR-T cell differentiation. (**A**–**C**) 20BBZ CAR-T cells and 20BBZ-Bcl-2 CAR-T cells at different culturing time points were analyzed by flow cytometry, and CD4^+^ and CD8^+^ CAR-T cell percentages were determined. Statistical significance was determined by paired t-test. Statistical significance was indicated. Representative results from one of three replicate experiments are shown (**A**). Cell surface expression of exhaustion-related markers programmed cell death protein 1 (PD-1), T cell immunoglobulin and mucin domain-3 (TIM-3), and lymphocyte-activation gene 3 (LAG-3) according to flow cytometry. Each dot represents one donor. Statistical significance was determined by paired t -test. Statistical significance was indicated. n.s (not significant). Representative results from four replicate experiments are shown (**B**). The relative proportions of naive T cells (CD45RA^+^CD45RO^−^CCR7^+^, TN), central memory T cells (CD45RA^−^CD45RO^+^CCR7^+^, TCM), effector memory T cells (CD45RA^−^CD45RO^+^CCR7^−^, TEM), and effector T cells (CD45RA^+^CD45RO^−^CCR7^−^, TEFF). Statistical significance was determined by paired t-test. Statistical significance was indicated. Each dot represents one donor. Pooled results from three replicate experiments are shown (**C**).

Bcl-2 has been reported to enhance T cell survival and promote memory T cell development [24,25]. We analyzed whether overexpression of Bcl-2 could affect the differentiation of CAR-T cells, focusing on CD8^+^ and CD4^+^ cell differentiation, memory status, and exhaustion of markers during the in vitro culture period. Interestingly, there was an increase in the portion of CD4^+^ cells among the 20BBZ-Bcl-2 CAR-T cells compared to that of the 20BBZ CAR-T cells after the first week of long-term culturing (Figure 5A and Figure S4). This suggested that the Bcl-2 signal predominantly affected the proliferation and/or the survival of CD4^+^ CAR-T cells over that of CD8^+^ CAR-T cells. To determine whether Bcl-2 signaling could affect the exhaustion status of CAR-T cells, we examined the exhaustion-related cell surface markers programmed cell death protein 1 (PD-1), T cell immunoglobulin and mucin domain-3 (TIM-3), and lymphocyte-activation gene 3 (LAG-3). We observed a slight decrease tendency in the expressions of LAG-3 and TIM-3 in CD8+ 20BBZ-Bcl-2 CAR-T cells compared with those in 20BBZ CAR-T cells (Figure 5B) after the fourth round of stimulation. Since Bcl-2 signaling can help preserve the central memory phenotype of T cells [26], we tested whether Bcl-2 signaling could affect the memory status of CAR-T in our newly designed construct. We found a slight increase tendency in the proportion of central memory T cells (CCR7^+^CD45RO^+^CD45RA^−^) and a slight decrease tendency in the proportion of effector memory T cells (CCR7^−^CD45RO^+^CD45RA^−^) in 20BBZ-Bcl-2 CAR-T cells during the culture period compared with those of 20BBZ CAR-T cells (Figure 5C).

### 2.6. 20BBZ-Bcl-2 CAR-T Cells Showed Better Anti-Tumor Effects In Vivo

**Figure 6:**
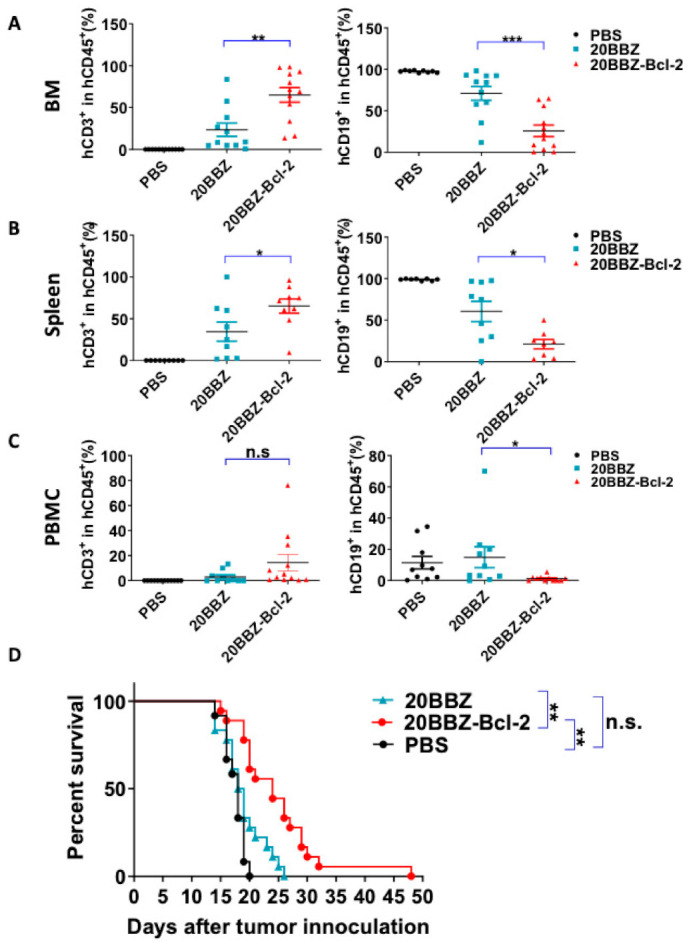
20BBZ-Bcl-2 CAR-T cells exhibit enhanced persistence and anti-tumor effects in vivo. (**A**–**C**) Immunodeficient NOD/SCID/γ−/− (NSG) mice were intravenously inoculated with 3 × 10^5^ Raji cells. The tumor-bearing mice were treated 7 days later with phosphate buffered saline (PBS), 1 × 10^7^ 20BBZ CAR-T cells, or 1 × 10^7^ 20BBZ-Bcl-2 CAR-T cells. Bone marrow, spleen, and peripheral blood were collected 7 days after treatment and analyzed for CAR-T cell (mCD45^−^hCD45^+^hCD3^+^) persistence and Raji (mCD45^−^hCD45^+^ hCD19^+^) tumor-cell burden in mCD45^−^hCD45^+^ population. Each point represents one mouse. Pooled results from two of three replicate experiments are shown. Statistical significance was determined by unpaired t-test. Statistical significance was presented by * *p* < 0.05, ** *p* < 0.01, *** *p* < 0.001 and n.s (not significant). (**D**) Kaplan–Meier analysis of the survival of mice. Each point represents one mouse. Pooled results from three replicate experiments are shown (**D**).

Our in vitro findings indicated that 20BBZ-Bcl-2 CAR-T cells exhibited enhanced proliferation ability. This led us to question whether this correlated with in vivo anti-tumor potency. To address this, we established a Raji lymphoma xenograft model in immunodeficient NOD/SCID/γ−/− (NSG) mice for evaluating the therapeutic efficacy of the 20BBZ-Bcl-2 CAR-T cells. We compared the remaining CAR-T cells in the peripheral blood, spleen, and bone marrow of the animals (Figure 6A–C and Figure S5). There were more CAR-T cells in the spleen and the bone marrow of the group of mice treated with 20BBZ-Bcl-2 CAR-T cells compared to that in the group treated with 20BBZ CAR-T cells. Consistent with the increased cell population of 20BBZ-Bcl-2 CAR-T cells, tumor burden in the peripheral blood, spleen, and bone marrow was lower in mice treated with 20BBZ-Bcl-2 CAR-T cells compared to that in the group treated with 20BBZ CAR-T cells (Figure 6A–C and Figure S5). Furthermore, Raji-tumor bearing mice treated with 20BBZ-Bcl-2 CAR T cells showed prolonged overall survival compared to that of the 20BBZ CAR-T cell treatment group (Figure 6D). Both 20BBZ CAR-T cell and 20BBZ-Bcl-2 CAR-T cell treated groups showed significantly longer survival time than that of the PBS control group. Taken together, these results indicated that the Bcl-2 enhanced the anti-tumor activity of CAR-T cells in vivo and prolonged survival of tumor-bearing mice.

## 3. Discussion

CAR-T cell immunotherapy has achieved remarkable efficacy for treating refractory and relapse hematological malignancies [27,28,29]; however, its efficacy in solid tumors is limited. Various approaches have been used to enhance CAR-T cell infiltration of solid tumors, persistence, and cytotoxicity. Manipulations of the intrinsic CAR design, including the single-chain variable fragment (scFv) affinity [30], the linker length [31], and the co-stimulation domain selection [9], have improved the anti-tumor activity of CAR-T cells. Other studies have included, beyond CAR, the addition of functional modules to overcome the limitations. These have included interleukin (IL)-7 and chemokine (C-C motif) ligand 19 (CCL19) to recruit endogenous anti-tumor immune cells [32], a PD-1-CD28 switch [33], and scFv-PD-1 secretion [34] to reduce immune suppression in the tumor microenvironment and Bispecific T cell engager (BiTE) secretion to prevent tumor heterogeneity-induced tumor escape [35]. In addition, some studies have expanded CAR-T cells by expressing IL-15 to preserve the persisting memory T cells [36].

Our current study focused on improving the persistence of CAR-T cells by promoting survival. The mechanism of T cell persistence has been intensively studied. Various pathways and molecules, such as SIRT6, survivin, and Bcl-2, can promote cell survival in unmodified T cells. However, Bcl-2 demonstrated the strongest anti-apoptotic ability in CAR-T cells compared to that of the others tested. One possibility is that the CAR-induced activation mechanism was different from that of the TCR-induced activation mechanism. Therefore, conclusions made regarding T cells in general may not be directly applicable to CAR-T cells. Several studies have demonstrated that Bcl-2 plays important anti-apoptosis roles in various cell types, including tumor cells [37], and Bcl-2 inhibition is a critical area of anti-cancer drug development [37]. Bcl-2 expression during T cell development is dynamically regulated, suggesting an important role in T cell maturation [38,39]. Mature Bcl-2-deficient T cells have shorter lifespans and are sensitive to apoptotic stimulation [16,40,41]. Consistent with these observations, overexpression of Bcl-2 significantly reduced background levels of apoptosis and AICD in CAR-T cells in vitro and promoted the survival of CAR-T cells in vivo. Importantly, there was a greater proportion of cells of the central memory subtype in the 20BBZ-Bcl-2 CAR-T cells compared to that in the 20BBZ-CAR-T cells. Central memory T cells have a better capacity to reconstitute the pool of memory T cells and to mediate protective immunity when compared with relatively short-lived effector memory T cells, which may have contributed to the beneficial increase in vitro survival during long-term culturing. Other Bcl-2 family proteins, such as Bcl-xl and MCL-1, have shown similar anti-apoptosis effects in other cell types [42,43]. It will be interesting to investigate whether Bcl-xl and MCL-1 could function similarly to Bcl-2 in CAR-T cells. Although the survival of CAR-T was enhanced, 20BBZ-Bcl-2 CAR-T cells showed similar cytotoxicity ability as that of 20BBZ CAR-T cells. It will be interesting to investigate whether the combination of Bcl-2 overexpression and other strategies, such as PD-1-CD28 switch and CD40L overexpression, will further improve the anti-tumor effector of CAR-T cells.

Bcl-2 can reduce apoptosis and promote survival of both CD4^+^ and CD8^+^ T cells. In our study, we used total T cells for the generation of our CAR-T cells and observed an increased CD4^+^/CD8^+^ ratio in the 20BBZ-Bcl-2 CAR-T cells compared with that in the 20BBZ CAR-T cells. This suggested that CD4^+^ and CD8^+^ CAR-T cell survivals were differentially regulated by Bcl-2. Interpretation of the differential role of Bcl-2 in CD4^+^ and CD8^+^ CAR-T cells may be aided by using purified CD4^+^ or CD8^+^ T cells in future studies.

One limitation of our study is that we did not test whether this approach is suitable for other solid tumor targets beyond CD20. It will be interesting to design solid-tumor-targeting CAR-T cells with Bcl-2 overexpression and to test their anti-tumor efficacy in solid tumor models in future investigations. Another limitation is the potential malignancy-inducing effect by Bcl-2. Bcl-2 is overexpressed in various types of cancer and is a key mediator for chemotherapy resistance [37]. An inducible death-switch, such as Herpes simplex virus thymidine kinase (HSV-TK) [44], inducible caspase-9 [45], or Rituximab binding epitope [46], will provide an alternative strategy for eliminating these cells if required.

In summary, we developed a novel CAR construct with constitutive expression of Bcl-2. This strategy provided the CAR-T cells with strong anti-apoptotic ability, enhanced CAR-T cell survival both in vitro and in vivo, and better anti-tumor efficacy in a xenograft tumor model. Our novel CAR design should be applicable to other CAR-T cells targeting various types of tumors and provides a new strategy for improving the efficacy of CAR-T cells.

## 4. Materials and Methods

### 4.1. Cell Lines

The Lenti-X 293T cell line was purchased from Clontech (Mountain View, CA, USA). The Raji cell line was purchased from the Chinese Academy of Sciences (Shanghai, China). Lenti-X 293T cells were cultured in Dulbecco’s Modified Eagle’s Medium (DMEM). Raji was maintained in -1640. All cell culture media were supplemented with 10% heat-inactivated fetal bovine serum (FBS) (Gibco, Paisley, UK), 2 mmol/L-glutamine, 100 units/mL penicillin, and 100 μg/mL streptomycin.

### 4.2. CAR Design and Lentivirus Production

CAR antigen-targeting regions (scFv) were derived from rituximab. The 20BBZ CAR consisted of the scFv being linked to the intracellular signaling domain containing 4-1BB and CD3ζ via the CD8α hinge and the transmembrane domain. Bcl-2, survivin, SIRT3, cFLIP, and SIRT6 were linked to CD3ζ using the self-cleaving porcine teschovirus-1 2A (P2A) peptide to generate anti-apoptotic molecules integrated into CAR. The CAR coding DNA was cloned into the pCDH-MSC-EF1 vector backbone (SBI System Biosciences, Palo Alto, CA) to generate a lentiviral transfer vector. The lentivirus was produced using Lenti-X 293T cells as previously described [47].

### 4.3. CAR-T Cell Generation

Peripheral blood mononuclear cells (PBMCs) were derived from cord blood provided by Shanghai Longyao Biotechnology Co., Ltd. (Shanghai, China) and were isolated using Ficoll-Paque density-gradient centrifugation. Total T cells were purified using an EasySep™ Human T Cell Isolation Kit (Stemcell). Purified T cells were seeded into 96-well plates and stimulated for 72 h with plate-bound anti-CD3 (0.25 μg/mL) and anti-CD28 (1 μg/mL) antibodies. Activated T cells were then transduced with lentivirus encoding the indicated CAR at a multiplicity of infection (MOI) of 10. During in vitro expansion, CAR-T cells were stimulated weekly with irradiated Raji cells (effector to target (E:T) = 3:1). CAR-T cells were cultured in RPMI-1640 medium supplemented with 10% heat-inactivated FBS, 2 mmol/L-glutamine, 100 units/mL penicillin, and 100 μg/mL streptomycin, 50 IU/mL IL-2, and 4 ng/mL IL-21.

### 4.4. In Vitro Killing Assay

A total of 1 × 10^5^ CAR-T cells were co-cultured with Raji cells in 96-well plates at effector to target ratios of 1:1, 1:2, and 1:4. Forty eight hours later, the cells were harvested and analyzed by flow cytometry. Anti-CD3 and anti-CD19 were used to distinguish CAR-T and tumor cells, respectively.

### 4.5. RT-qPCR

Total RNA was extracted using an E.Z.N.A.^®^ Total RNA Kit I (Omega Bio-Tek, Norcross, GA, USA.). The RNA was reverse transcribed using ReverTra Ace reverse transcriptase (Toyobo, New York, NY, USA), and gene expression of the specified genes was quantified by real time PCR using KOD SYBR qPCR mix (Toyobo) according to manufacturer’s instructions.

### 4.6. In Vivo Anti-Tumor Activity of CAR-T Cells

Female NOD/SCID/γ−/− (NSG) mice were purchased from the Shanghai Model Organisms Center, Inc. (Shanghai, China). All mice were maintained under specific pathogen-free conditions. Animal care and use were in accordance with institutional and National Institutes of Health (NIH) protocols and guidelines. All studies were approved by the Animal Care and Use Committee of Shanghai Jiao Tong University (ethic code: A2015019, approved on 25 June 2015).

Mice were injected intravenously (i.v.) with 3 × 10^5^ Raji cells. One week after tumor-cell inoculation, the mice were randomly grouped and treated with PBS, 1 × 10^7^ 20BBZ CAR-T cells, or 1 × 10^7^ 20BBZ-Bcl-2 CAR-T cells. One week post injection with CAR-T cells, the percentages of CAR-T cells and Raji cells in the peripheral blood were assessed by flow cytometry. Nine days after CAR-T cell injection, the mice were sacrificed, and the tumor burden and the CAR-T cell persistence in bone marrow and spleen were analyzed by flow cytometry.

### 4.7. Flow Cytometry

Single-cell suspensions of cells were incubated with anti-CD16/32 (anti-FcγRII/III, clone 2.4G2) for 10 min and then subsequently stained with the indicated fluorescently labeled monoclonal antibodies (Abs). Anti-human CD45RO^−^APC-A700 (UCHL1), anti-human CD45RA^−^PB450 (HI100), anti-human CD62L-FITC (DREG-56), anti-human CD4-PB450 (OKT4), anti-human CD8α-APC-A750 (HIT8a), anti-human CD3-FITC (OKT3), and anti-mouse CD45-PB450 (30-F11) Abs were purchased from Biolegend. Anti-human PD-1-APC (eBioJ105), anti-human TIM-3-PE (F35-2E2), anti-human LAG-3-FITC (3DS223H), anti-human CD19^−^FITC (HIB19), anti-human CD3-FITC (OKT3), anti-human CD45-APC (HI30), and anti-human CD19^−^FITC (HIB19) Abs were purchased from eBioscience. The anti-human CCR-7-APC (552176) Ab was purchased from BD Bioscience, and the goat anti-mouse IgG and F(ab’)2- FITC Ab was purchased from Jackson Immuno Research. Samples were analyzed using a Cytoflex Flow Cytometer (Beckman Coulter, Brea, CA, USA), and the data were analyzed using FlowJo software v.10.4. (TreeStar, Inc., San Carlos, CA, USA).

### 4.8. Statistical Analysis

Statistical analyses were performed using GraphPad Prism version 8.0 software. Significance of the in vitro assays was determined by a two-sided Student’s unpaired *t*-test. A two-sided log rank test was applied to assess mouse survival. Where indicated, * *p* < 0.05, ** *p* < 0.01, and *** *p* < 0.001 were considered statistically significant results.

## 5. Conclusions

Integrating anti-apoptotic molecule Bcl-2 into CAR design is a useful strategy for improving CAR-T cell proliferation in vitro and anti-tumor activity in vivo, which provides a potential approach for optimization of CAR-T cells against lymphoma in clinic.

## Data Availability

The data presented in this study are available on request from the corresponding author.

## Supplementary Materials

The following are available online at https://www.mdpi.com/2072-6694/13/2/197/s1, Figure S1: Comparison of long-term proliferation of 20BBZ CAR-T cells and 20BBZ CAR-T cells with the indicated anti-apoptotic molecules, Figure S2: Comparison of long-term proliferation of 20BBZ CAR-T cells and 20BBZ-Bcl-2 CAR-T cells, Figure S3: The mRNA expression levels of the indicated anti-apoptotic genes in 20BBZ-Bcl-2 CAR-T cells were analyzed by RT-qPC, Figure S4: 20BBZ CAR-T cells and 20BBZ-Bcl-2 CAR-T cells at different culturing time points were analyzed by flow cytometry and CD4^+^ CAR-T cell percentages were determined, Figure S5: Immunodeficient NOD/SCID/γ−/− (NSG) mice were intravenously inoculated with 3 × 10^5^ Raji cells.
