# Supplementary material for: Bcl-2 Enhances Chimeric Antigen Receptor T Cell Persistence by Reducing Activation-Induced Apoptosis
Source: Cancers (Basel). 2021 Jan 8;13(2):197. doi: 10.3390/cancers13020197 (PMC7827522; doi:10.3390/cancers13020197)
Supplement: Supplementary file 1 [file cancers-13-00197-s001.pdf]

Supplementary Materials: Bcl-2 Enhances Chimeric Antigen Receptor T Cell Persistence by Reducing Activation-Induced Apoptosis

Haiyong Wang, Ping Han, Xinyue Qi, Fanlin Li, Min Li , Lilv Fan, Huihui Zhang, Xiaoqing Zhang and Xuanming Yang

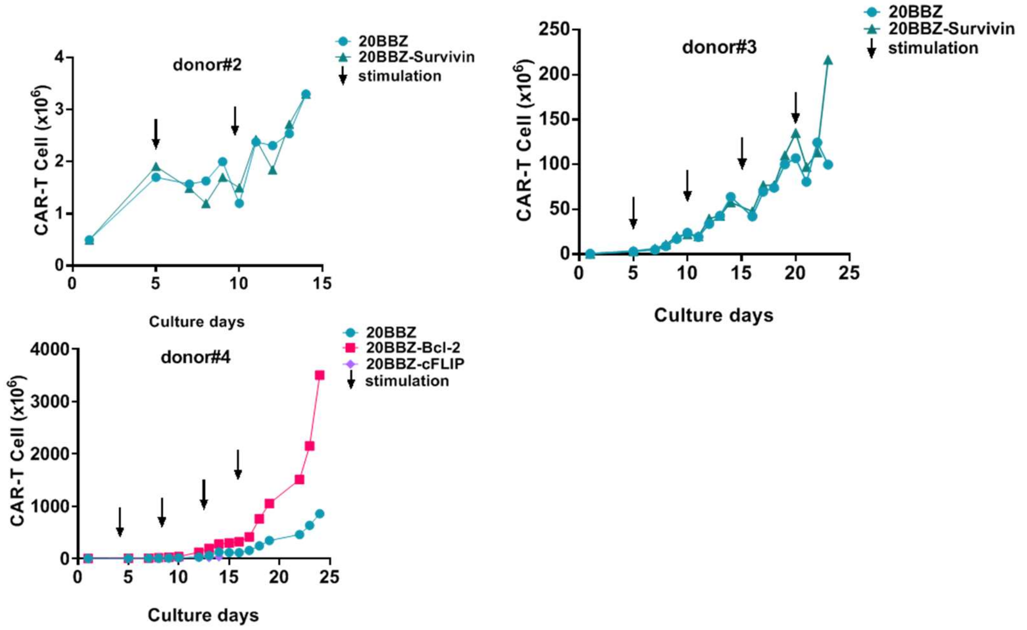

Figure S1. Comparison of long-term proliferation of 20BBZ CAR-T cells and 20BBZ CAR-T cells with the indicated anti-apoptotic molecules. The arrows indicated the irradiated-Raji stimulation.

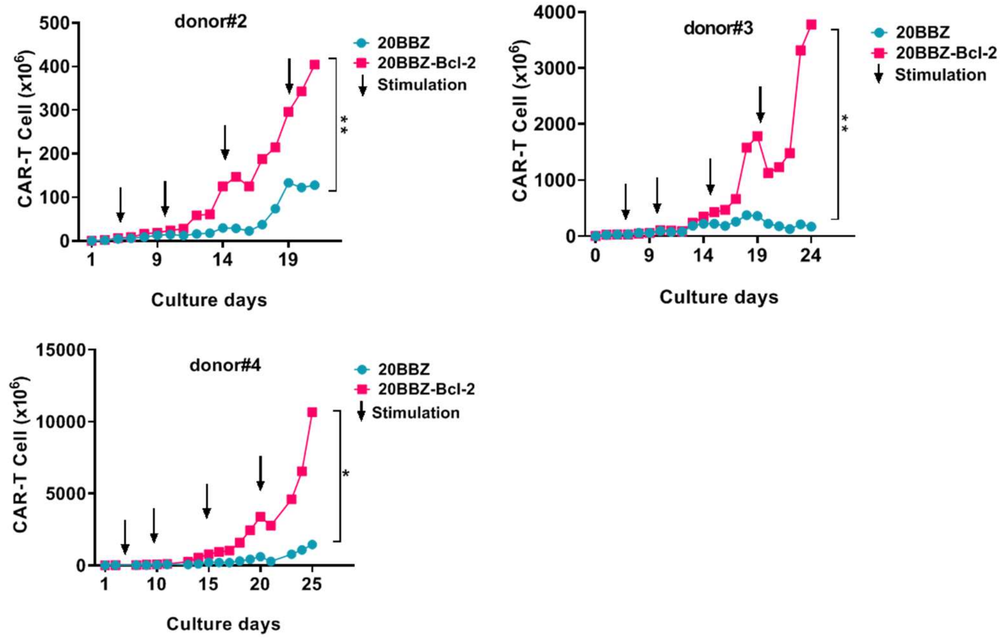

Figure S2. Comparison of long-term proliferation of 20BBZ CAR-T cells and 20BBZ-Bcl-2 CAR-T cells. The arrows indicated the irradiated-Raji stimulation. Statistical significance was determined by unpaired t-test. Statistical significance was presented by * $p < 0.05$, ** $p < 0.01$.

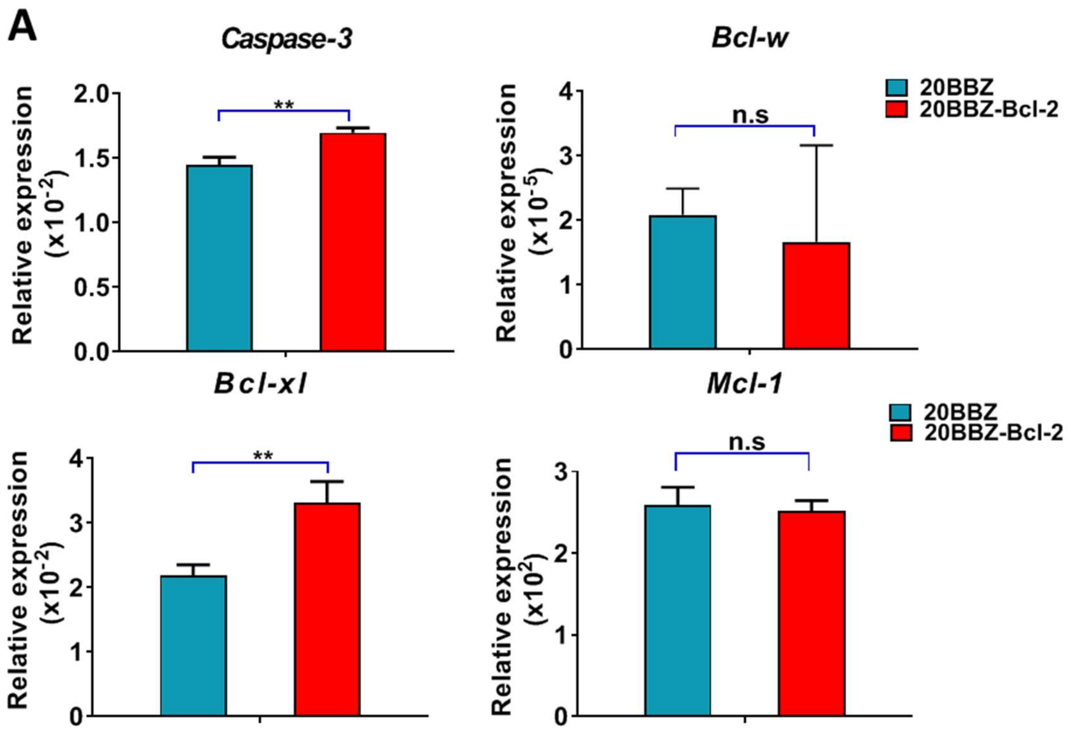

Figure S3. The mRNA expression levels of the indicated anti-apoptotic genes in 20BBZ-Bcl-2 CAR-T cells were analyzed by RT-qPCR. Statistical significance was determined by unpaired t-test. Statistical significance was presented by ** $p < 0.01$ and n.s (not significant).

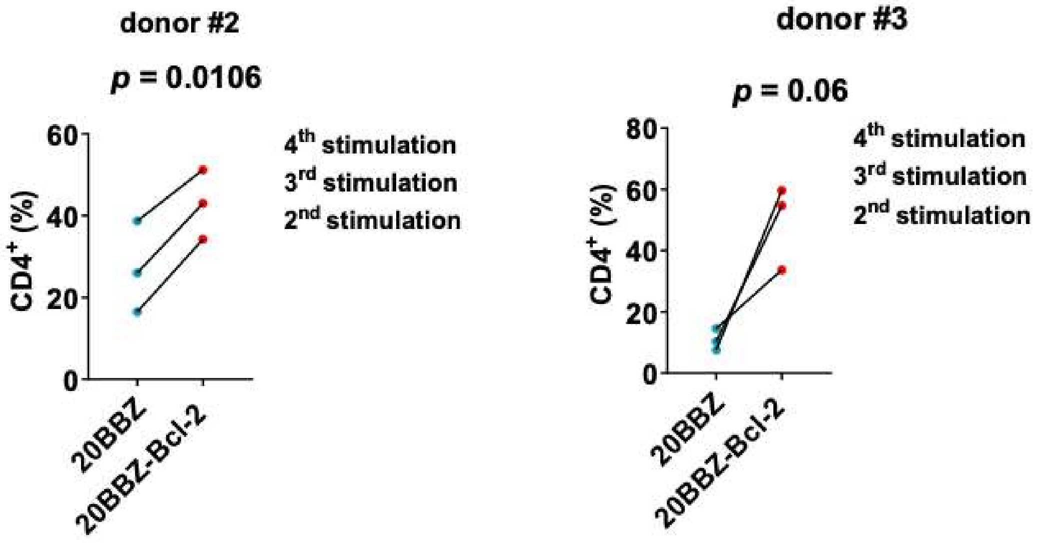

Figure S4. 20BBZ CAR-T cells and 20BBZ-Bcl-2 CAR-T cells at different culturing time points were analyzed by flow cytometry and $CD4^+$ CAR-T cell percentages were determined.

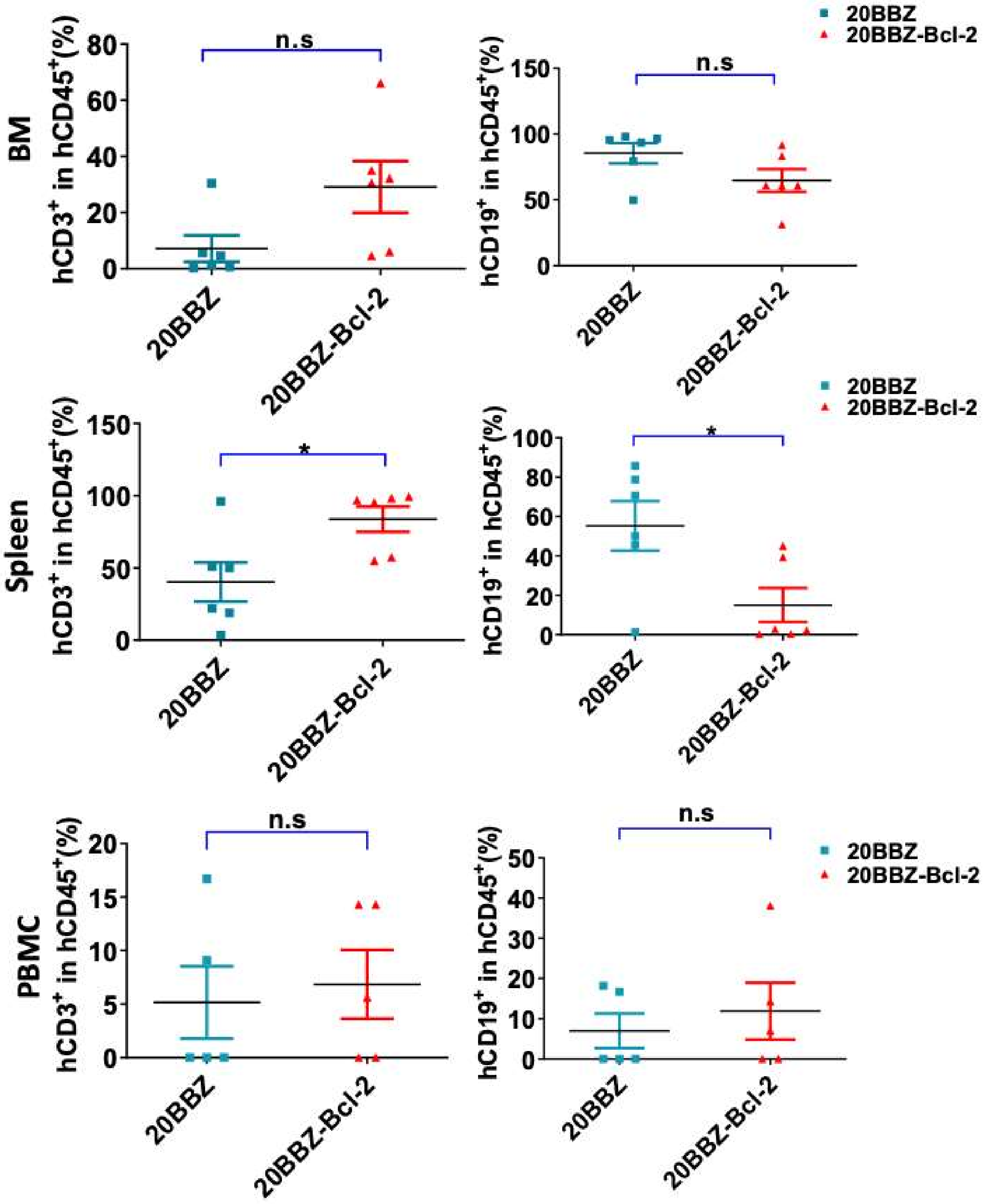

Figure S5. Immunodeficient NOD/SCID/γ−/− (NSG) mice were intravenously inoculated with 3 × 10^5 Raji cells. The tumor-bearing mice were treated 7 d later with 1 × 10^7 20BBZ CAR-T cells, or 1 × 10^7 20BBZ-Bcl-2 CAR-T cells. Bone marrow, spleen, and peripheral blood were collected 7 d after treatment and analyzed for CAR-T cell (mCD45−hCD45+hCD3+) persistence and Raji (mCD45−hCD45+ hCD19+) tumor-cell burden in mCD45-hCD45+ population. Statistical significance was determined by unpaired t-test. Statistical significance was presented by * $p < 0.05$ and n.s (not significant).

© 2020 by the authors. Licensee MDPI, Basel, Switzerland. This article is an open access article distributed under the terms and conditions of the Creative Commons Attribution (CC BY) license (http://creativecommons.org/licenses/by/4.0/).
